# Supplementary material for: Mapping of in vivo cleavage sites uncovers a major role for yeast RNase III in regulating protein-coding genes
Source: bioRxiv. 2025 Aug 13:2025.03.07.642061. Preprint. [Version 2] doi: 10.1101/2025.03.07.642061 (PMC12363893; doi:10.1101/2025.03.07.642061)
Supplement: Supplement 1 [file NIHPP2025.03.07.642061v2-supplement-1.pdf]

bioRxiv preprint doi: https://doi.org/10.1101/2025.03.07.642061; this version posted August 13, 2025. The copyright holder for this preprint (which was not certified by peer review) is the author/funder, who has granted bioRxiv a license to display the preprint in perpetuity. It is made available under aCC-BY 4.0 International license.

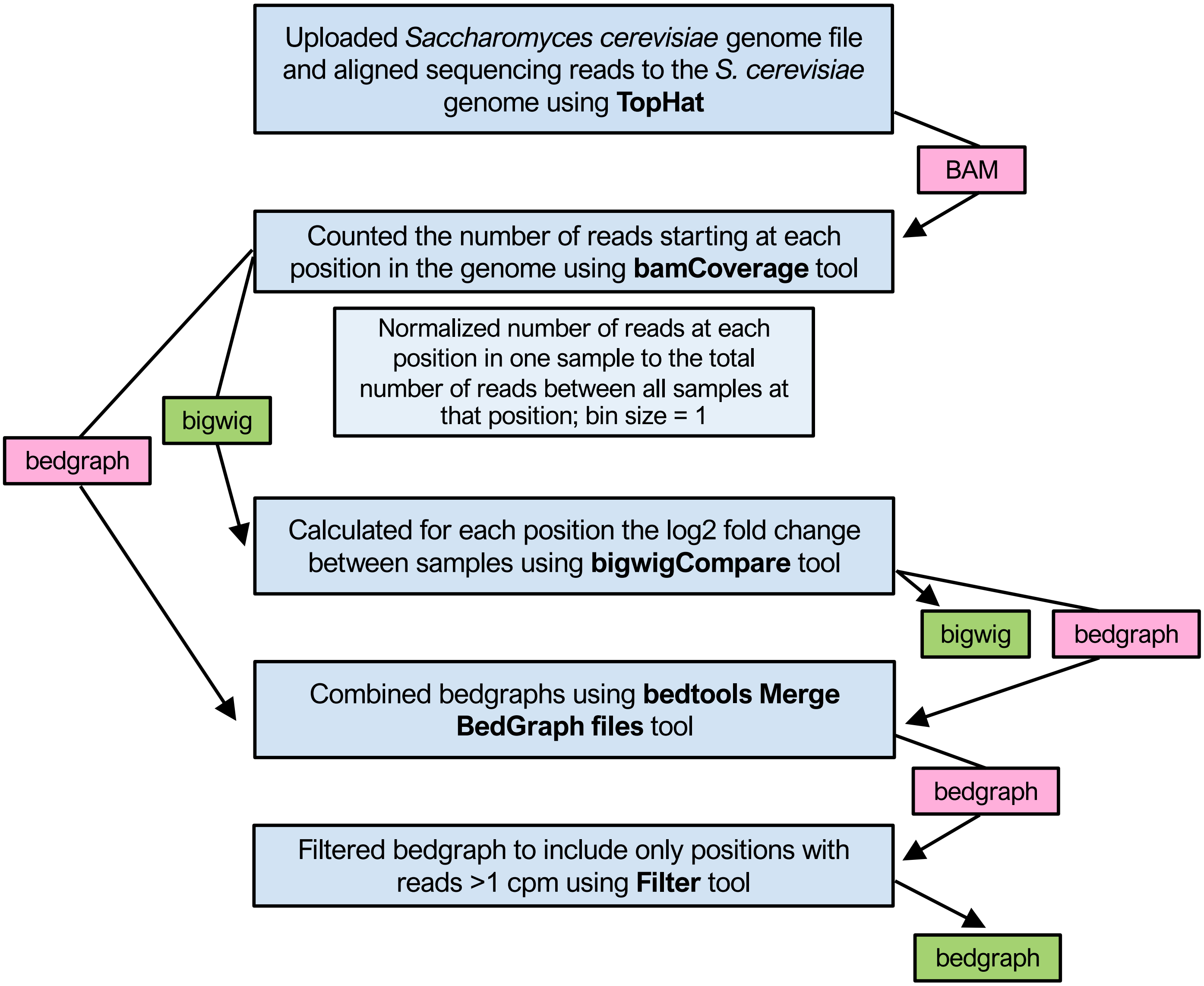

Supplemental Figure 1: Bioinformatic pipeline for analysis of PARE data in Galaxy. Pink boxes represent files used only for a subsequent step in the pipeline. Green boxes represent files used for further analysis in Microsoft Excel or Integrative Genomics Viewer (IGV).

bioRxiv preprint doi: https://doi.org/10.1101/2025.03.07.642061; this version posted August 13, 2025. The copyright holder for this preprint (which was not certified by peer review) is the author/funder, who has granted bioRxiv a license to display the preprint in perpetuity. It is made available under aCC-BY 4.0 International license.

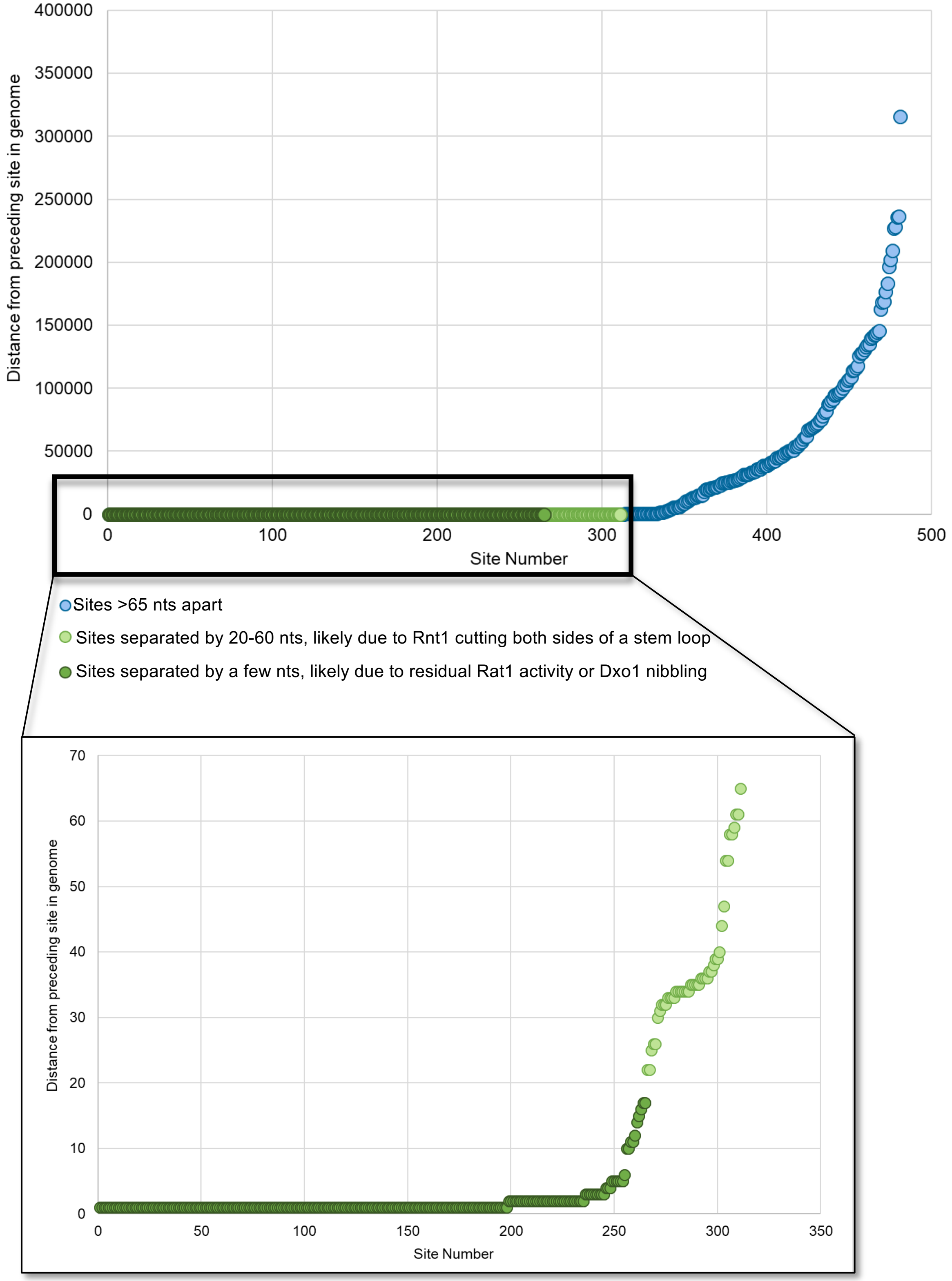

Supplemental Figure 2: Rnt1 cleavage site distribution. 496 Rnt1 cleavage sites are clustered into 166 putative substrates. Hits separated by 1 nt are likely due to residual Rat1 and/or Dxo1 activity, and hits separated by 20-60 nts are likely due to Rnt1 cutting both sides of a stem loop.

bioRxiv preprint doi: https://doi.org/10.1101/2025.03.07.642061; this version posted August 13, 2025. The copyright holder for this preprint (which was not certified by peer review) is the author/funder, who has granted bioRxiv a license to display the preprint in perpetuity. It is made available under aCC-BY 4.0 International license.

Supplemental Figure 3

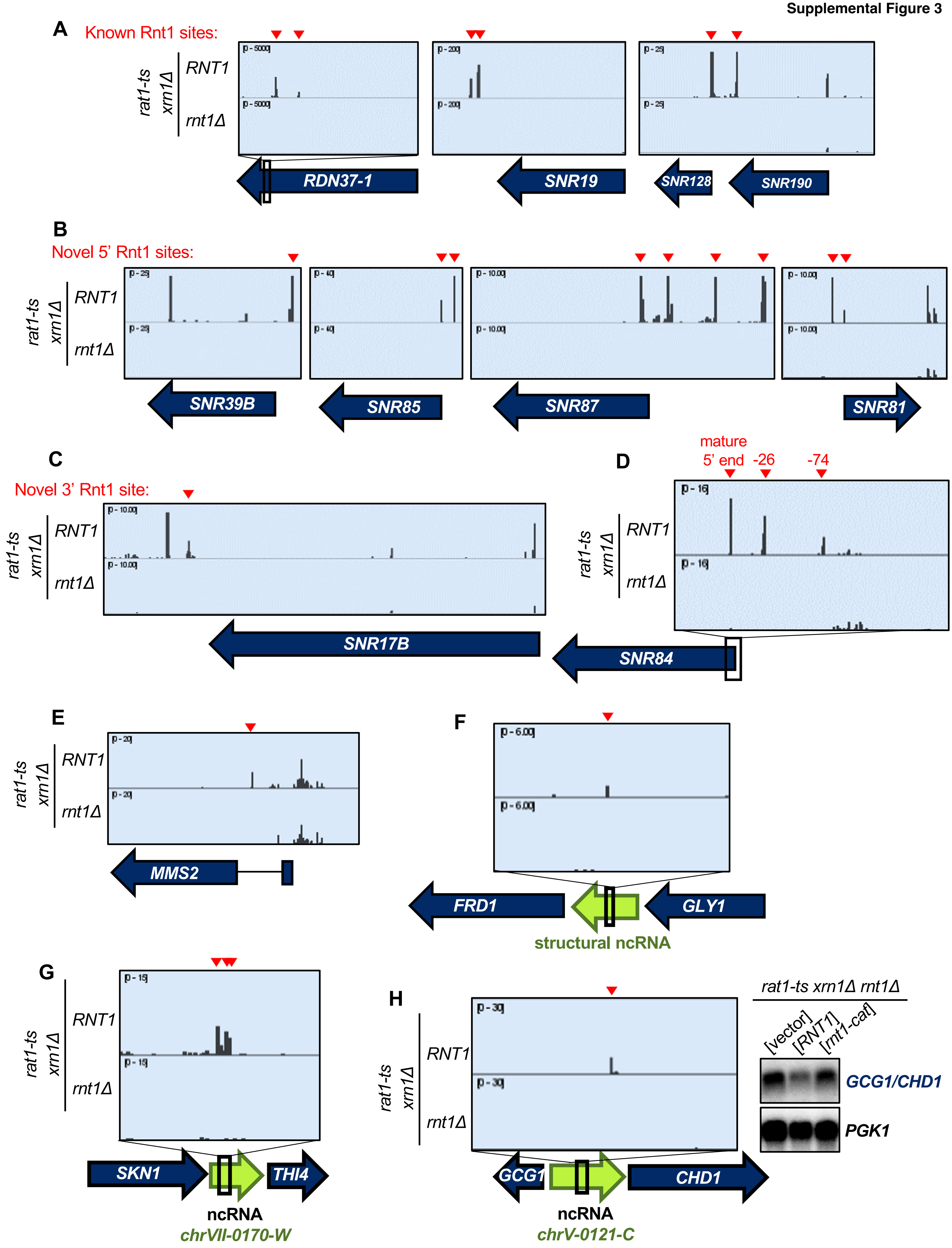

bioRxiv preprint doi: https://doi.org/10.1101/2025.03.07.642061; this version posted August 13, 2025. The copyright holder for this preprint (which was not certified by peer review) is the author/funder, who has granted bioRxiv a license to display the preprint in perpetuity. It is made available under aCC-BY 4.0 International license.

Supplemental Figure 3: **(A)** Examples of known Rnt1 cleavage sites detected by PARE in ncRNAs: pre-rRNA 3' ETS, pre-U1 snRNA, and pre-*SNR190/128* snoRNA dicistronic transcript. **(B)** Novel Rnt1 cleavage sites detected by PARE at the 5' ends of snoRNAs *SNR39B*, *SNR85*, *SNR87*, and *SNR81*. **(C)** Novel Rnt1 cleavage sites detected by PARE at the 3' end of the U3 snRNA (*SNR17B*). **(D)** PARE detects a Rnt1 cleavage site at the mature 5' end of *SNR84* as well as sites 26 and 74 nts upstream of the mature end, suggesting Rnt1 cleavage of a 5' extension upstream of pre-*SNR84*. **(E)** Example of an intron lacking a snoRNA that is still cleaved by Rnt1. **(F-H)** Rnt1 cleavage sites detected by PARE in intergenic regions: **(F)** between *FRD1* and *GLY1* contain a structural ncRNA; **(G)** between *SKN1* and *THI4* contain the uncharacterized ncRNA *chrVII-0170-W*; **(H)** between *GCG1* and *CHD1* contain the uncharacterized ncRNA *chrV-0121-C*. Probing the *GCG1*/*CHD1* intergenic region by northern blot showed RNA stabilization in the absence of a catalytically active Rnt1. Red arrowheads, Rnt1 cleavage sites.

bioRxiv preprint doi: https://doi.org/10.1101/2025.03.07.642061; this version posted August 13, 2025. The copyright holder for this preprint (which was not certified by peer review) is the author/funder, who has granted bioRxiv a license to display the preprint in perpetuity. It is made available under aCC-BY 4.0 International license.

Supplemental Figure 4

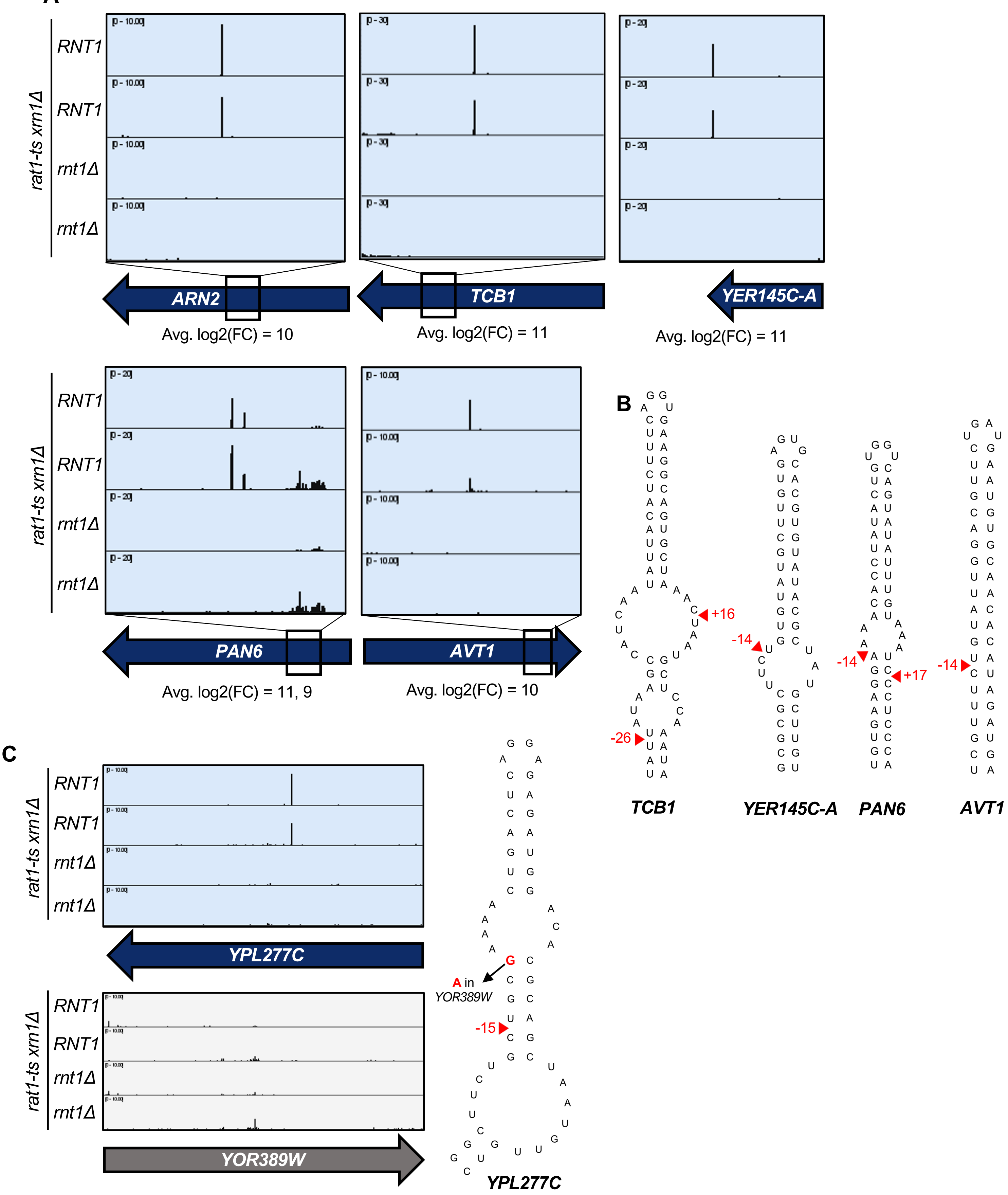

bioRxiv preprint doi: https://doi.org/10.1101/2025.03.07.642061; this version posted August 13, 2025. The copyright holder for this preprint (which was not certified by peer review) is the author/funder, who has granted bioRxiv a license to display the preprint in perpetuity. It is made available under aCC-BY 4.0 International license.

Supplemental Figure 4: (A) *ARN2* is a published mRNA target detected by PARE. Other newly identified and highly cleaved Rnt1 mRNA targets detected by PARE include *TCB1*, *YER145C-A*, *PAN6*, and *AVT1*. **(B)** The predicted secondary structures of novel Rnt1 mRNA targets in (A), with Rnt1 cleavage sites indicated by red arrowheads. **(C)** Rnt1 cleavage site detected in *YPL277C* but not in *YOR389W* presumably because of a single nt difference in the *YPL277C* stem loop that stabilizes the structure.

bioRxiv preprint doi: https://doi.org/10.1101/2025.03.07.642061; this version posted August 13, 2025. The copyright holder for this preprint (which was not certified by peer review) is the author/funder, who has granted bioRxiv a license to display the preprint in perpetuity. It is made available under aCC-BY 4.0 International license.

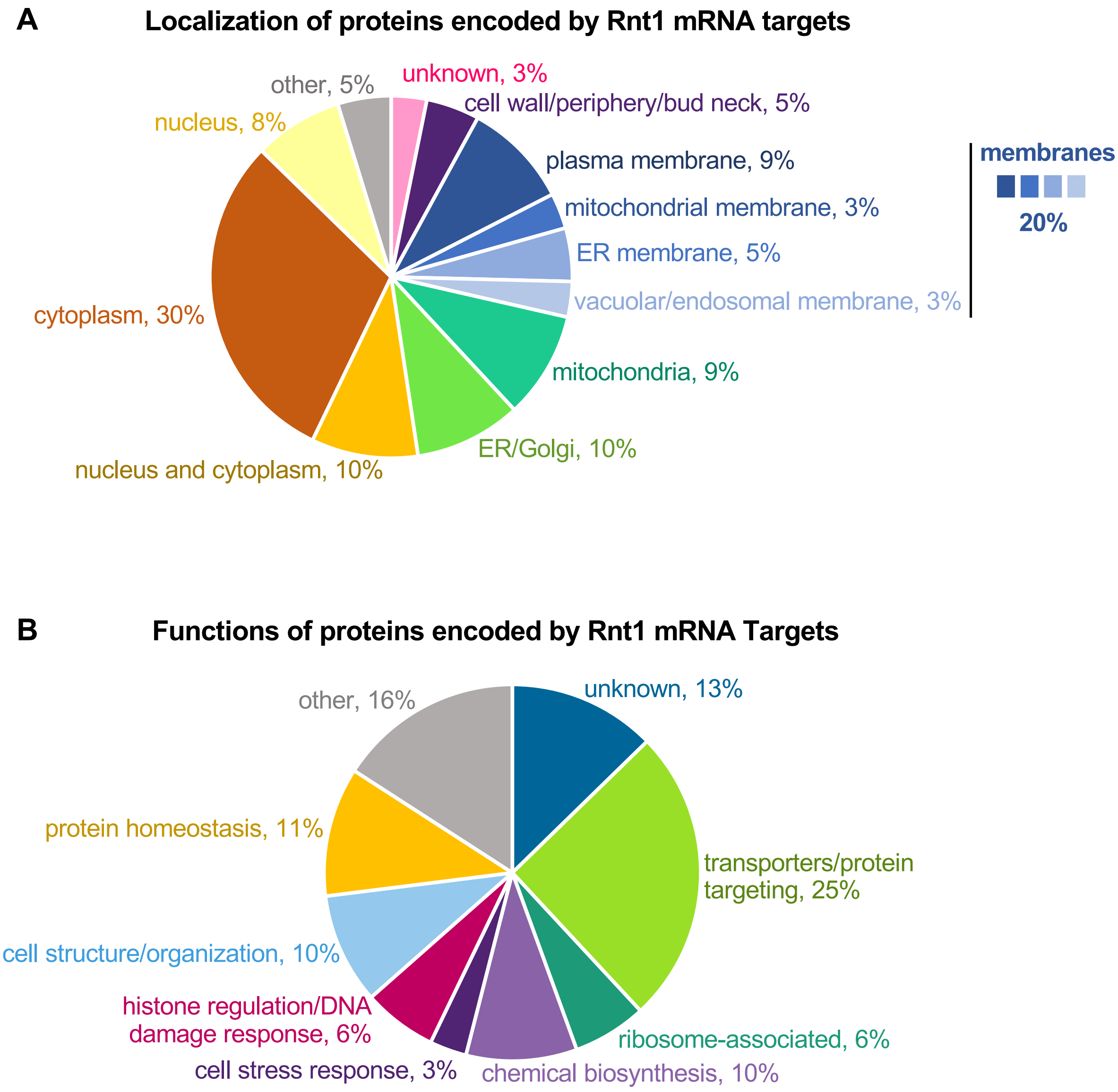

Supplemental Figure 5: (A) Rnt1-cleaved mRNAs encode proteins with varying subcellular localization. **(B)** Rnt1-cleaved mRNAs encode proteins that carry out various cellular functions.

bioRxiv preprint doi: https://doi.org/10.1101/2025.03.07.642061; this version posted August 13, 2025. The copyright holder for this preprint (which was not certified by peer review) is the author/funder, who has granted bioRxiv a license to display the preprint in perpetuity. It is made available under aCC-BY 4.0 International license.

Supplemental Figure 6

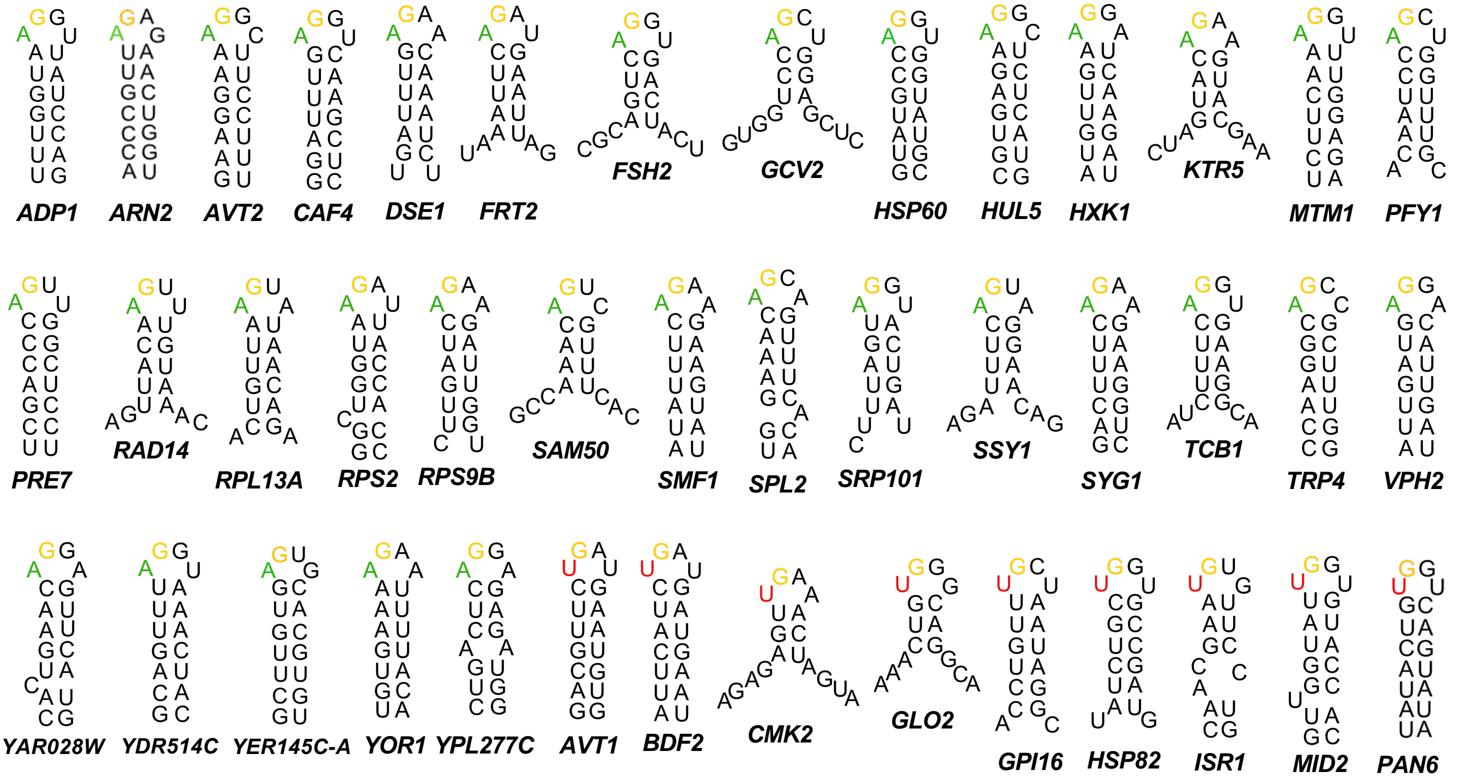

Supplemental Figure 6: Predicted mRNA tetraloop sequences plus surrounding sequences used for alignment in Figure 2F.

bioRxiv preprint doi: https://doi.org/10.1101/2025.03.07.642061; this version posted August 13, 2025. The copyright holder for this preprint (which was not certified by peer review) is the author/funder, who has granted bioRxiv a license to display the preprint in perpetuity. It is made available under aCC-BY 4.0 International license.

Supplemental Figure 7

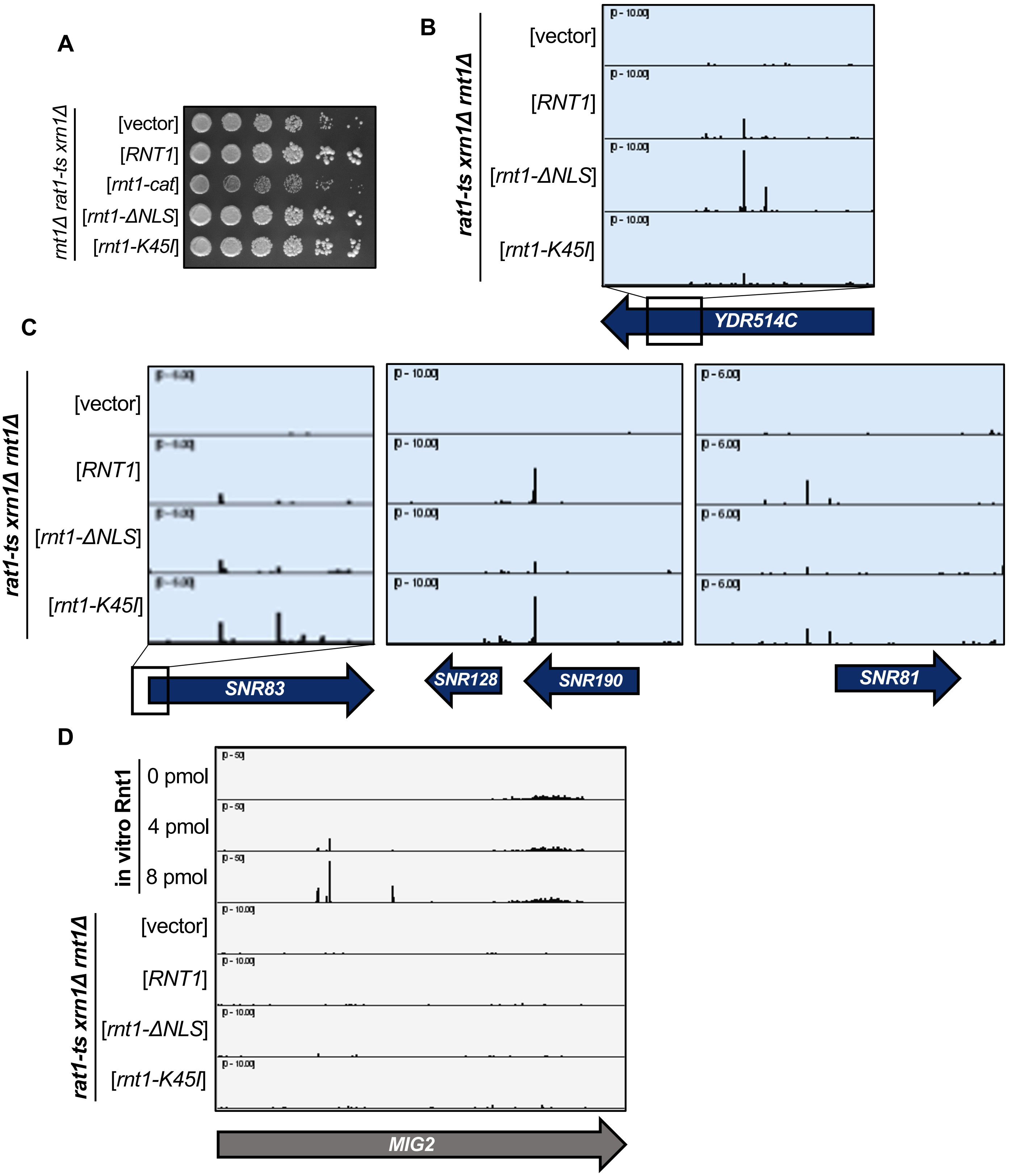

Supplemental Figure 7: (A) The *rnt1-ΔNLS* and *rnt1-K45I* mutants grow similarly to a strain containing wild-type *RNT1* expressed from a plasmid. **(B)** *YDR514C* is cleaved more efficiently in the *rnt1-ΔNLS* strain compared to *RNT1*. **(C)** Rnt1 ncRNA targets *SNR83*, *SNR190/128*, and *SNR81* are cleaved more efficiently in the *rnt1-K45I* mutant compared to the *RNT1* strain, and less efficiently in the *rnt1-ΔNLS* strain compared to *RNT1*. **(D)** Although *MIG2* is cleaved in in vitro, no PARE peaks are detected for in vivo cleavage, even in the *rnt1-ΔNLS* strain.

bioRxiv preprint doi: https://doi.org/10.1101/2025.03.07.642061; this version posted August 13, 2025. The copyright holder for this preprint (which was not certified by peer review) is the author/funder, who has granted bioRxiv a license to display the preprint in perpetuity. It is made available under aCC-BY 4.0 International license.

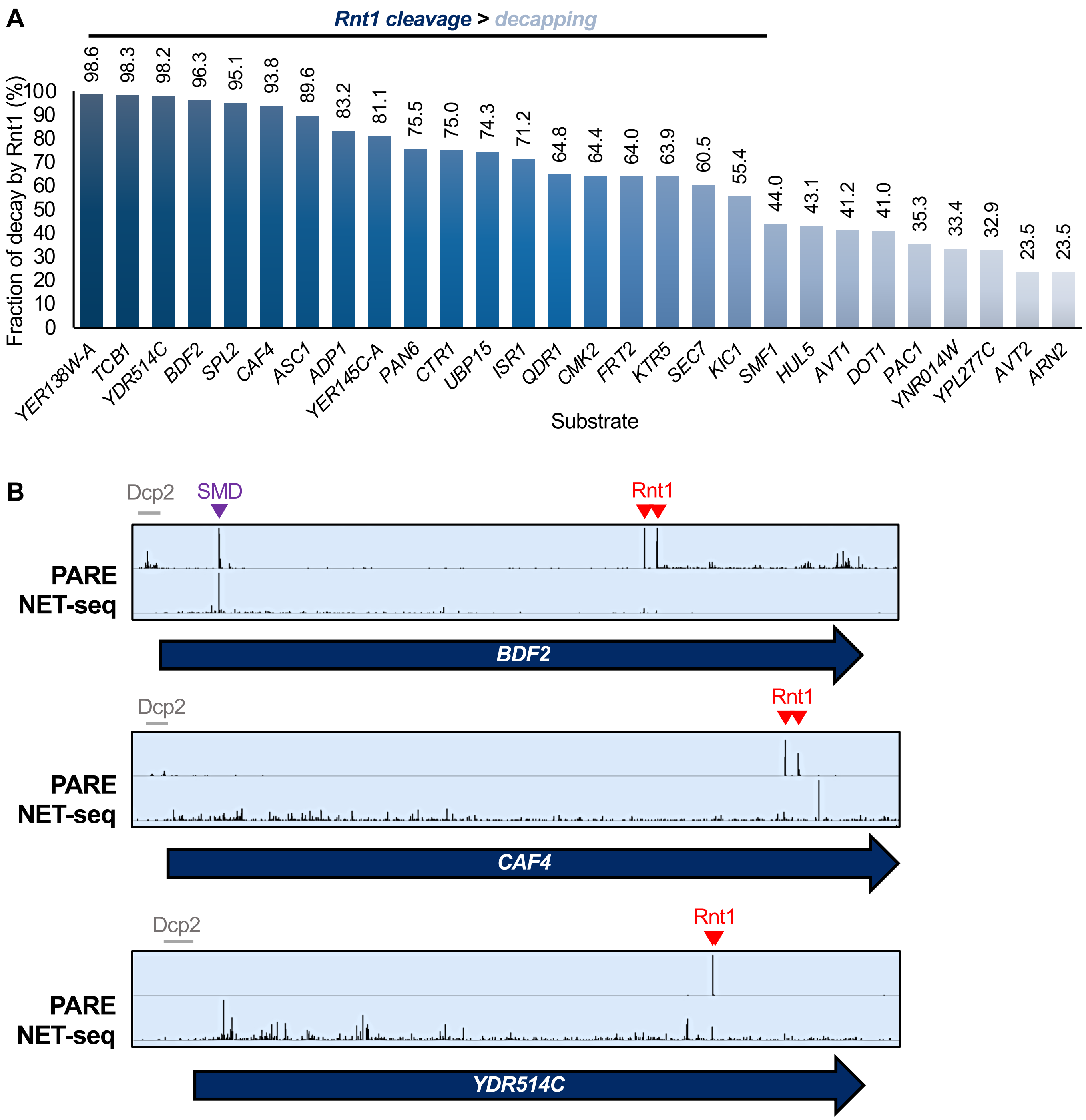

Supplemental Figure 8: Rnt1 preferentially cleaves polyadenylated mRNA. **(A)** Rnt1 cleavage products predominate over decapping products in poly(A)$^+$ PARE. For each mRNA substrate identified as an Rnt1 target, the fraction of the degradome generated by Rnt1 was calculated as the frequency of Rnt1 cleavage products divided by the total amount of products resulting from both Rnt1 and decapping. These values were calculated from PARE peak height and represent the averages of two biological replicates. **(B)** NET-seq, which sequences mRNA 3' OH ends still associated with RNA polymerase II, identifies the spliceosome-mediated decay (SMD) product of *BDF2* (purple arrowhead) but does not identify prominent peaks corresponding to Rnt1 cleavage (red arrowheads), suggesting that Rnt1 cleaves after cleavage and polyadenylation and release from RNA polymerase II. Grey bars, decapping peaks.

bioRxiv preprint doi: https://doi.org/10.1101/2025.03.07.642061; this version posted August 13, 2025. The copyright holder for this preprint (which was not certified by peer review) is the author/funder, who has granted bioRxiv a license to display the preprint in perpetuity. It is made available under aCC-BY 4.0 International license.

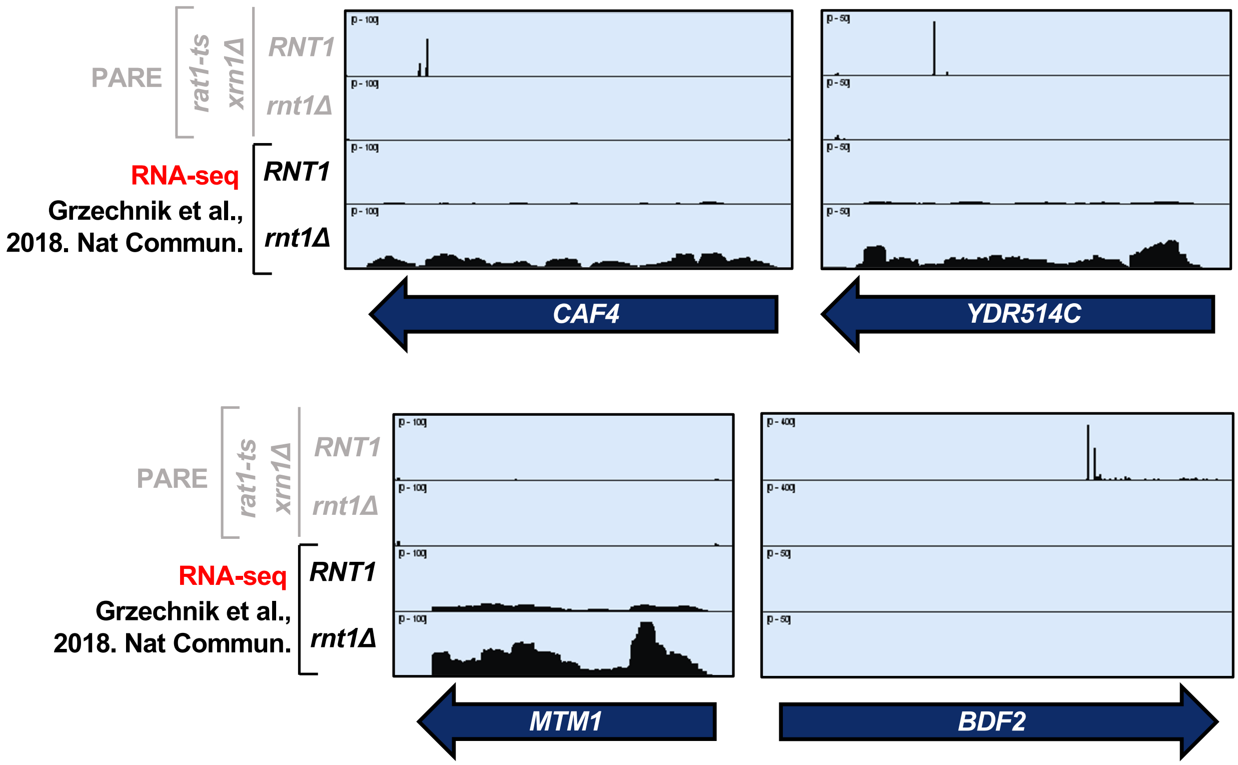

Supplemental Figure 9: Rnt1 PARE data generated in this study (top two panels), compared to one replicate of *RNT1* vs *rnt1Δ* RNA-seq data previously published by Grzechnik et al., 2018 (bottom two panels). Rnt1 may affect the gene expression levels of some mRNA targets such as *CAF4*, *YDR514C*, and *MTM1*, but not of others like *BDF2*.

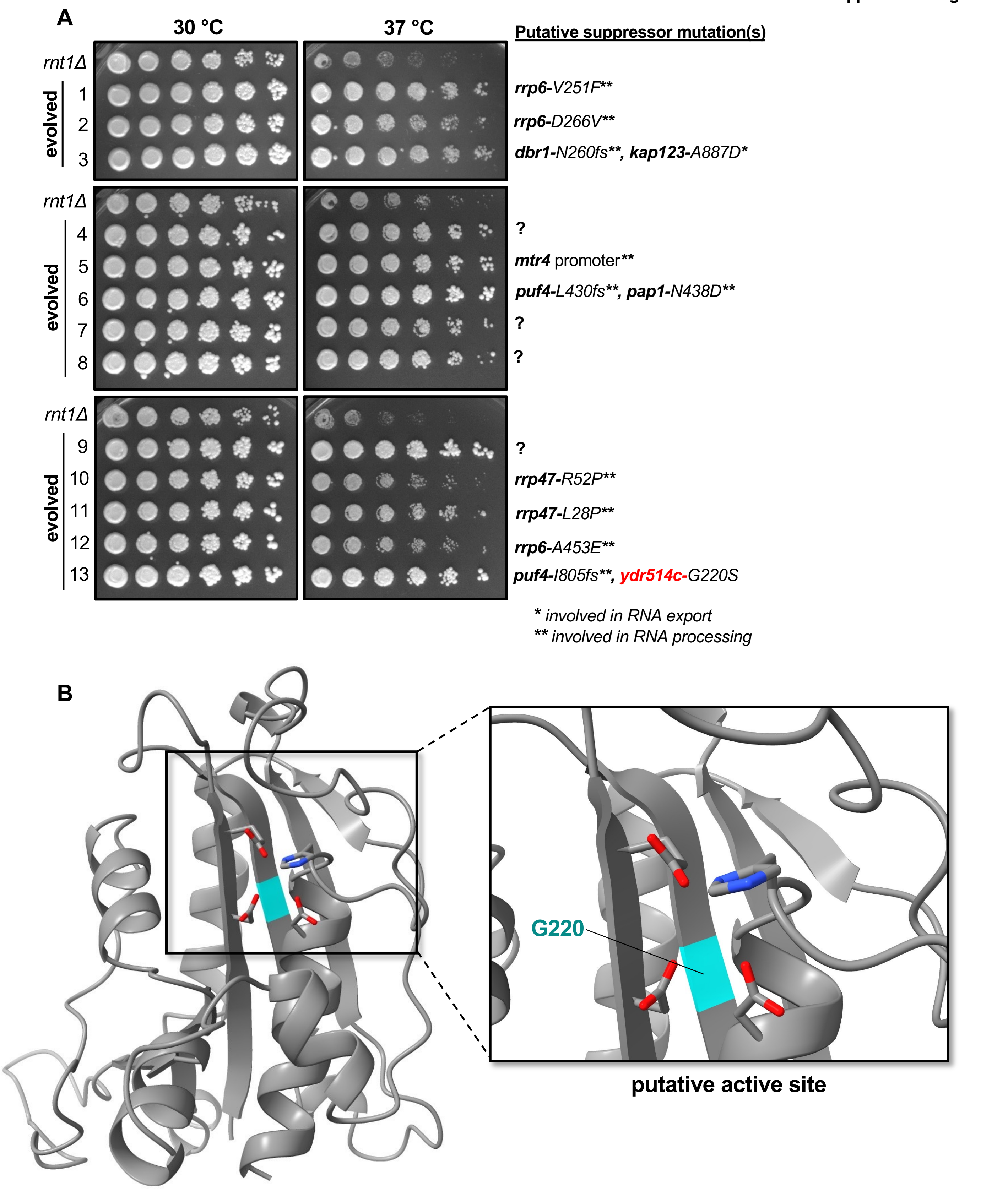

Supplemental Figure 10: (A) Evolved *rnt1Δ* strains grow better than the *rnt1Δ* parent strain at both 30 °C and 37 °C. Mutations identified by whole-genome sequencing are shown on the right. **(B)** AlphaFold-predicted structure of the Ydr514c nuclease domain (left) and putative active site (right). Red, oxygens; blue, nitrogen; cyan, glycine 220 which was mutated to serine in evolved *rnt1*Δ strain 13.

Table S2: List of plasmids used in this study:

Plasmid	Description	Source
pAG32	hphMX4, conferring resistance to Hygromycin B	Goldstein & McCusker, 1999
pRS415	CEN empty vector with *LEU2* gene	Sikorski & Hieter, 1989
pAV1595	CEN pRS315 containing GFP under native *RNT1* promoter, *LEU2*	Catala et al., 2004
pAV1596	CEN pRS315 containing *RNT1* CDS (residues 1-471) with N-terminal GFP tag, under native *RNT1* promoter, *LEU2*	Catala et al., 2004
pAV1597	CEN pRS315 containing *RNT1* CDS (residues 1-471) with *D245R* mutation and N-terminal GFP tag, under native *RNT1* promoter, *LEU2*	Catala et al., 2004
pAV412	CEN pRS315 containing C-terminal-truncated *RNT1* CDS (residues 1-463) with N-terminal GFP tag, under native *RNT1* promoter, *LEU2*	Catala et al., 2004
pAV413	CEN pRS315 containing *RNT1* CDS (residues 1-471) with *K45I* mutation and N-terminal GFP tag, under native *RNT1* promoter, *LEU2*	Catala et al., 2004
p425GPD	2μ p425GPD empty vector, LEU2	Mumberg et al., 1995
pAV2084	2μ p425GPD YDR514C under GPD promoter, LEU2	This study
pAV2085	2μ p425GPD YDR514C-SL* under GPD promoter, LEU2	This study

Table S3: List of yeast strains used in this study:

Name	Genotype	Source
BY4742	*MATα*, *ura3Δ-0*, *leu2Δ-0*, *his3Δ-1*, *lys2Δ-0*, *MET15*	Baker Brachmann et al., 1998
yAV1085	*MATα*, *ura3Δ-0*, *leu2Δ-0*, *his3Δ-1*, *lys2Δ-0*, *met15Δ-0*, *rnt1Δ::NEO*	This study
yAV4514	*MATa*, *ura3Δ-0*, *leu2Δ-0*, *his3Δ-1*, *lys2Δ-0*, *met15Δ-0*, *rnt1Δ::HYG*	This study
N.A.	*MATa, ura3Δ-0, leu2Δ-0, his3Δ-200, lys2Δ-0, rnt1Δ::HIS3*	Catala et al., 2012
yAV5012	*MATα*, *ura3Δ-0*, *leu2Δ-0*, *his3Δ-1*, *lys2Δ-0*, *met15Δ-0*, *rnt1Δ::NEO [LEU2]*	This study
yAV4419	*MATα*, *ura3Δ-0*, *leu2Δ-0*, *his3Δ-1*, *lys2Δ-0*, *met15Δ-0*, *rnt1Δ::NEO [GFP, RNT1, LEU2]*	This study
yAV4421	*MATα*, *ura3Δ-0*, *leu2Δ-0*, *his3Δ-1*, *lys2Δ-0*, *met15Δ-0*, *rnt1Δ::NEO [GFP, rnt1-ΔNLS, LEU2]*	This study
yAV4422	*MATα*, *ura3Δ-0*, *leu2Δ-0*, *his3Δ-1*, *lys2Δ-0*, *met15Δ-0*, *rnt1Δ::NEO [GFP, rnt1-K45I, LEU2]*	This study
yAV3484	*MATa*, *ura3Δ-0*, *leu2Δ-0*, *his3Δ-1*, *LYS2*, *MET15*, *rat1-ts::URA3*, *can1 Δ::LEU2*, *MFApr-HIS3*	Kofoed et al., 2015
yAV3248	*MATα*, *ura3Δ-0*, *leu2Δ-0*, *his3Δ-1*, *lys2Δ-0*, *MET15*, *xrn1Δ::HYG*	Hurtig et al., 2021
yAV4752	*MATα*, *ura3Δ-0*, *leu2Δ-0*, *his3Δ-1*, *LYS2*, *MET15*, *puf4Δ::NEO*	This study
yAV4754	*MATα*, *ura3Δ-0*, *leu2Δ-0*, *his3Δ-1*, *LYS2*, *MET15*, *ydr514cΔ::NEO*	This study
yAV4212	*MATα*, *ura3Δ-0*, *leu2Δ-0*, *his3Δ-1*, *lys2Δ-0*, *met15Δ-0*, *rat1-ts::URA3*, *MFApr-HIS3*, *xrn1Δ::HYG*	This study
yAV4954	*MATα*, *ura3Δ-0*, *leu2Δ-0*, *his3Δ-1*, *LYS2*, *met15Δ-0*, *rnt1Δ::HYG*, *puf4Δ::NEO*	This study
yAV4956	*MATα*, *ura3Δ-0*, *leu2Δ-0*, *his3Δ-1*, *lys2Δ-0*, *met15Δ-0*, *rnt1Δ::HYG*, *ydr514cΔ::NEO*	This study
yAV4054	*MATα*, *ura3Δ-0*, *leu2Δ-0*, *his3Δ-1*, *lys2Δ-0*, *met15Δ-0*, *rnt1Δ::NEO*, *rat1-ts::URA3*, *MFApr-HIS3*, *xrn1Δ::HYG*	This study
yAV4070	*MATα*, *ura3Δ-0*, *leu2Δ-0*, *his3Δ-1*, *lys2Δ-0*, *met15Δ-0*, *rnt1Δ::NEO*, *rat1-ts::URA3*, *xrn1Δ::HYG [GFP, LEU2]*	This study
yAV4071	*MATα*, *ura3Δ-0*, *leu2Δ-0*, *his3Δ-1*, *lys2Δ-0*, *met15Δ-0*, *rnt1Δ::NEO*, *rat1-ts::URA3*, *xrn1Δ::HYG [GFP, RNT1, LEU2]*	This study

yAV4072	*MATα*, *ura3Δ-0*, *leu2Δ-0*, *his3Δ-1*, *lys2Δ-0*, *met15Δ-0*, *rnt1Δ::NEO*, *rat1-ts::URA3*, *xrn1Δ::HYG [GFP, rnt1-D245R, LEU2]*	This study
yAV4193	*MATα*, *ura3Δ-0*, *leu2Δ-0*, *his3Δ-1*, *lys2Δ-0*, *met15Δ-0*, *rnt1Δ::NEO*, *rat1-ts::URA3*, *xrn1Δ::HYG [GFP, rnt1-ΔNLS, LEU2]*	This study
yAV4194	*MATα*, *ura3Δ-0*, *leu2Δ-0*, *his3Δ-1*, *lys2Δ-0*, *met15Δ-0*, *rnt1Δ::NEO*, *rat1-ts::URA3*, *xrn1Δ::HYG [GFP, rnt1-K45I, LEU2]*	This study

Table S4: List of oligos used in this study:

Oligo	Description	Sequence
oAV3246	*SNR83* probe	GCAACTACGGTAATTGGTCC
oAV3486	*BDF2* probe	TGCCTTCCTACGCAAAAGCG
oAV3233	*CAF4* probe	CCTCGTTTGCGCCAACAGTG
oAV3525	*PGK1* probe	CGGAACCTGGGGCAGAAGCC
oAV3566	*GCG1/CHD1* probe	AACGAGAGGGTAACAGTATG
oAV3939	YDR514C_SDM_F	AGAAAATTTATAGCATGTGTTATGGCAAGAAGGGTTCATCCCTCGGTAAACTACTAAGGCTTTTTCACTTGCCACAT
oAV3940	YDR514C_SDM_R	ATGTGGCAAGTGAAAAAGCCTTAGTAGTTTACCGAGGGATGAACCCTTCTTGCCATAACACATGCTATAAATTTTCT
oAV3949	YDR514C_F_SpeI_overlap	agtttcgacggattctagaaATGAGTAGCTCCACAAAGTGCA
oAV3950	YDR514C_R_SalI_overlap	gacataactaattacatgactcgaggTCACACAAGAGTACTTTTGAAGGC
